# *POWERDRESS* and Diversified Expression of the *MIR172* Gene Family Bolster the Floral Stem Cell Network

**DOI:** 10.1371/journal.pgen.1003218

**Published:** 2013-01-17

**Authors:** Rae Eden Yumul, Yun Ju Kim, Xigang Liu, Ruozhong Wang, Junhui Ding, Langtao Xiao, Xuemei Chen

**Affiliations:** 1 Department of Botany and Plant Sciences, Institute of Integrative Genome Biology, University of California Riverside, Riverside, California, United States of America; 2 ChemGen IGERT program, Center for Plant Cell Biology, Institute of Integrative Genome Biology, University of California Riverside, Riverside, California, United States of America; 3 College of Bioscience and Biotechnology, Hunan Agricultural University, Changsha, China; 4 Howard Hughes Medical Institute, University of California Riverside, Riverside, California, United States of America; The University of North Carolina at Chapel Hill, United States of America

## Abstract

Termination of the stem cells in the floral meristem (also known as floral determinacy) is critical for the reproductive success of plants, and the molecular activities regulating floral determinacy are precisely orchestrated during the course of floral development. In *Arabidopsis thaliana*, regulators of floral determinacy include several transcription factor genes, such as *APETALA2* (*AP2*), *AGAMOUS* (*AG*), *SUPERMAN* (*SUP*), and *CRABSCLAW* (*CRC*), as well as a microRNA (miRNA), miR172, which targets *AP2*. How the transcription factor and miRNA genes are coordinately regulated to achieve floral determinacy is unknown. A mutation in *POWERDRESS* (*PWR*), a previously uncharacterized gene encoding a SANT-domain-containing protein, was isolated in this study as an enhancer of the weakly indeterminate *ag-10* allele. *PWR* was found to promote the transcription of *CRC*, *MIR172a*, *b*, and *c* and/or enhance Pol II occupancy at their promoters, without affecting *MIR172d* or *e*. A mutation in mature miR172d was additionally found to enhance the determinacy defects of *ag-10* in an *AP2*-dependent manner, providing direct evidence that miR172d is functional in repressing *AP2* and thereby contributes to floral determinacy. Thus, while *PWR* promotes floral determinacy by enhancing the expression of three of the five *MIR172* members as well as *CRC*, *MIR172d*, whose expression is *PWR*-independent, also functions in floral stem cell termination. Taken together, these findings demonstrate how transcriptional diversification and functional redundancy of a miRNA family along with *PWR*-mediated co-regulation of miRNA and transcription factor genes contribute to the robustness of the floral determinacy network.

## Author Summary

microRNAs (miRNAs) are 20–24 nucleotide RNAs that play regulatory roles in many developmental processes in plants and animals. Some miRNAs are encoded by multi-member gene families, and the members may exhibit differential expression patterns. However, the basis of this expression diversification and its developmental impact are poorly understood. By studying miR172, which represses its target *APETALA2* (*AP2*) and thereby promotes the determinate growth of flowers (also known as floral determinacy), we show that the five *MIR172* genes undergo differential transcriptional regulation. POWERDRESS (PWR), a previously uncharacterized SANT-domain-containing protein, promotes floral determinacy by enhancing the expression of *MIR172a-c*. *MIR172d*, whose expression is *PWR*-independent, was found to be functional in floral determinacy by repressing *AP2*. *PWR* also promotes floral determinacy through a transcription factor previously implicated in this process. Thus, transcriptional diversification of a miRNA family and *PWR*-mediated co-regulation of miRNA and transcription factor genes involved in floral determinacy contribute to the robustness of this developmental network.

## Introduction

Flowers are a key adaptation of the angiosperm lineage that helps ensure reproductive success. At a particular point in floral development, stem cells in the floral meristem are terminated, thereby preventing the continual growth that typifies the shoot and root apical meristems of plants. In *Arabidopsis thaliana*, the floral homeotic gene *AGAMOUS* (*AG*) is required for both reproductive organ specification and floral determinacy. *AG*'s role in floral determinacy involves the negative regulation of the stem-cell-promoting gene *WUSCHEL* (*WUS*) in the floral meristem, via direct and indirect mechanisms [1]–[3].

Other known regulators of floral determinacy represent a wide range of functions and include signaling proteins, transcription factors, and microRNAs. Of particular importance for the present study are the YABBY transcription factor CRABSCLAW (CRC) and the floral homeotic transcription factor APETALA2 (AP2). *CRC*'s dual functions in determinacy and carpel development are notably similar to those of *AG*, and the subtly indeterminate *crc-1* mutant background has helped uncover the involvement of a number of other genes in floral determinacy [4]–[6]. *AP2* encodes an AP2-domain-containing transcription factor and is regulated by miR172. A role for *AP2* in maintaining stem cell fate was first proposed in the shoot apical meristem [7]. A similar role in the floral meristem was revealed when miR172-resistant *AP2* was expressed and found to confer indeterminate floral organ production [8]. This finding also suggested an indirect role for miR172, which limits the accumulation of AP2 protein by translational inhibition [9], in negatively regulating *WUS* to confer floral determinacy.

miRNAs, regulatory molecules 20–24 nucleotide in length, play critical roles in various aspects of plant development through sequence-specific regulation of their targets [10]–[12]. In plants, genes encoding miRNAs (*MIR* genes) typically reside between protein-coding genes and are transcribed by RNA polymerase II (Pol II) [13], [14]. Deeply conserved plant *MIR* genes typically belong to families with multiple members [15], [16]. Evidence from several *MIR* gene families in plants indicates that their evolution has features in common with the evolution of protein-coding gene families. For example, analysis of the *MIR156*, *MIR159*, and *MIR166* families revealed differences in the spatial and temporal expression of genes within these families, which suggests that expression diversification occurred after gene duplication [17]. What is less clear, however, is how diversification in the expression of individual family members specifically contributes to the developmental processes regulated by the mature miRNA species produced by a given family.

miR172 regulates AP2 transcription factor genes involved in the distinct processes of flowering time and floral development [18], but the individual contributions of the five *MIR172* genes to these processes are unknown. Three unique mature miR172 sequences are produced from these five loci (one from *MIR172a* and *b*, one from *MIR172c* and *d*, and one from *MIR172e*), and publicly available datasets indicate that the three mature miR172 species differentially accumulate in inflorescences, rosette leaves, seedlings, and siliques [19]. Furthermore, specific regulation of *MIR172c* and *MIR172e* in the outermost floral whorl by a transcriptional corepressor indicates that the *MIR172* family members are differentially regulated in the flower [9], [20], [21].

The complex interplay of both *MIR172* and transcription factor genes in the termination of the floral stem cells makes this developmental paradigm well suited for the investigation of how individual *MIR* family members contribute to the functions broadly assigned to the mature miRNA species. In the present study, the *POWERDRESS* (*PWR*) gene was found to promote floral determinacy through *CRC* and miR172. *PWR* promoted the transcription and/or enhanced Pol II occupancy at the promoters of *MIR172a*, *b*, and *c* but did not affect *MIR172d* or *e*. Coincidentally, a mutation in *MIR172d* was also found to enhance the determinacy defects of a weak *ag* allele. Taken together, these findings indicate that differential regulation of *MIR172* genes with overlapping functions enhances the robustness of the genetic network underlying floral stem cell termination.

## Results

### *PWR* is required for the proper termination of floral stem cells

**Figure 1:**
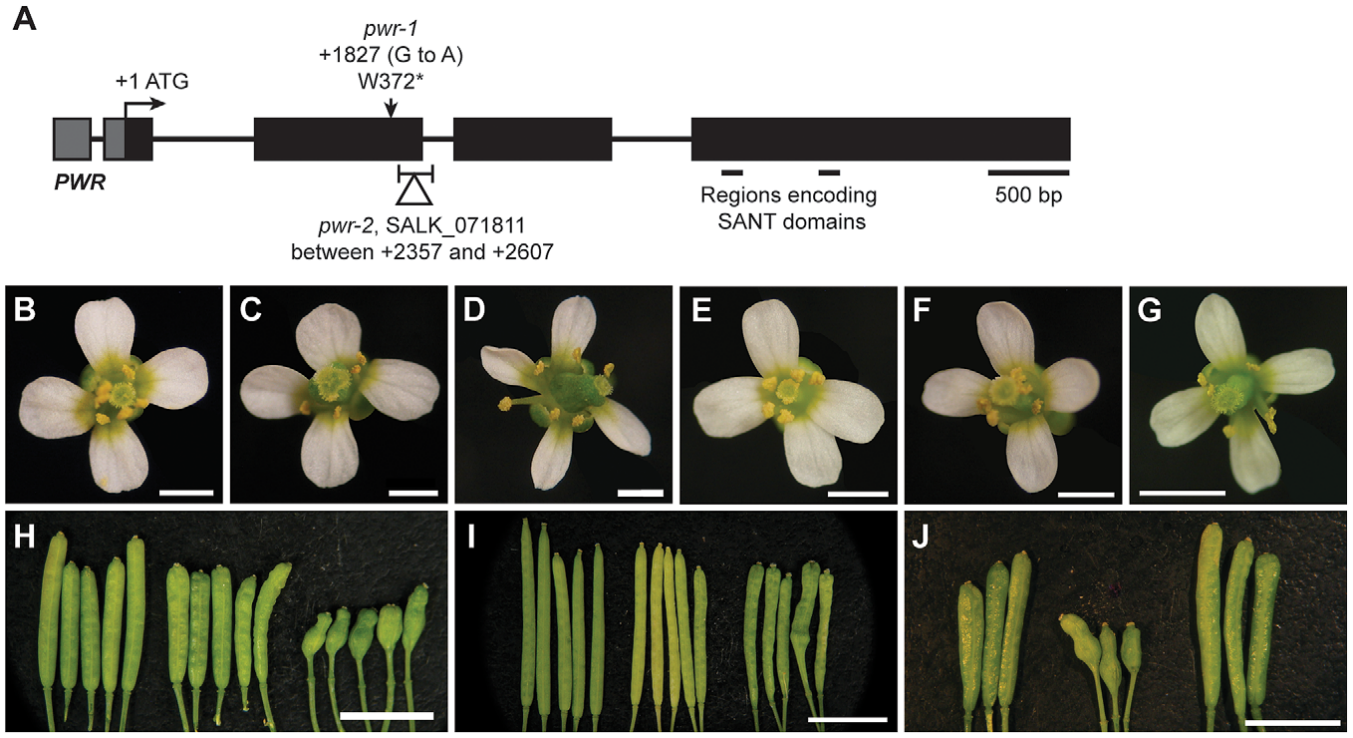
Gene diagram of *PWR* and phenotypes of *ag-10*, *ag-10 pwr-1*, *ag-10^Col^*, and *ag-10^Col^ pwr-2*. (A) Schematic diagram indicating the position of the G-to-A mutation in *pwr-1* that produces a premature stop codon and the approximate location of the T-DNA insertion in *pwr-2*. The regions encoding the putative SANT domains are also indicated. (B) Wild-type L*er* flower. (C) *ag-10* flower with slightly bulged carpels. (D) *ag-10 pwr-1* flower with narrow, slightly folded petals and a shortened and bulged gynoecium. (E) Wild-type Col flower. (F) *ag-10^Col^* flower. Col and *ag-10^Col^* flowers are indistinguishable. (G) *ag-10^Col^ pwr-2* flower with slightly aberrant carpels. (H) From left to right, siliques of L*er*, *ag-10*, and *ag-10 pwr-1*. *ag-10 pwr-1* has visible gynophores bearing shortened and bulged gynoecia containing internal flowers. (I) From left to right, siliques of Col, *ag-10^Col^*, and *ag-10^Col^ pwr-2*. Although most *ag-10^Col^ pwr-2* siliques have very subtle carpel defects, some are visibly bulged and contain additional floral organs inside. (J) Rescue analysis for *ag-10 pwr-1*. From left to right, *ag-10*, *ag-10 pwr-1*, and *pPWR:PWR-GFP* in the *ag-10 pwr-1* background. Scale bars = 500 bp in (A), 5 mm in (H) to (J), and 1 mm in all other panels.

To identify genes that regulate the termination of the stem cells in the floral meristem, an ethyl methanesulfonate screen was performed in the *ag-10* background, as previously reported [3], [22]. In contrast to the null *ag-1* mutant, which produces sterile flowers that indeterminately produce sepals and petals [23], the weak *ag-10* mutant has normal floral organ specification and slightly bulged gynoecia that very rarely, if at all, contain additional floral organs inside (Figure 1C, 1H; Table 1) [22]. Typical genetic enhancers isolated from the *ag-10* screen included those with consistently bulged and shortened siliques. In one of these mutants, elongated gynophores (i.e., the structure supporting the gynoecium) were observed along with the presence of ectopic floral organs in all of the dissected gynoecia, indicating a consistent enhancement of the floral determinacy defect of *ag-10* (Figure 1D, 1H; Table 1). The mutant also showed a small but statistically significant increase in floral organ number in the inner two whorls (Table 1). Map-based cloning revealed a G-to-A mutation that introduced a premature stop codon at the 372nd residue of a previously uncharacterized gene, At3g52250 (Figure 1A). The gene model for At3g52250 predicts a 1,656 amino acid protein with two SANT/Myb domains and putative DNA-binding and transcription factor activity (http://arabidopsis.org). SANT domains have high structural similarity to Myb DNA-binding domains but have been characterized as histone-binding domains important for chromatin remodeling activity in both plants and animals [24], [25]. The gene was subsequently named *POWERDRESS* (*PWR*) (based on the single mutant phenotype, described below), and the *ag-10* enhancer mutation was designated *pwr-1*. The floral determinacy defects of *ag-10 pwr-1* were rescued by transformation with an 8.1-kb genomic *PWR* fragment. As with *ag-10* plants, the flowers from five independent *pPWR:PWR-GFP* lines in the *ag-10 pwr-1* background rarely produced gynoecia containing ectopic floral organs, and elongated gynophores were not observed (Figure 1J).

**Table 1:** Floral organ counting and analysis of bulged carpel phenotypes.

Genotype	Sepals	Petals	Stamen	Carpels	Internal Organs (%)^1^	Gynoecium Length/Width^2^	N
*ag-10*	4±0	4±0	5.44±0.76	2.06±0.24	0%	5.16±1.19	50
*ag-10 pwr-1*	4±0	4±0	5.72±0.61*	2.34±0.69**	100%	2.17±0.38**	50
*ap2-2 ag-10 pwr-1*	NA^3^	NA	ND^4^	2.42±0.50	42%	4.07±0.99**	50
*ap2-2 ag-10*	NA	NA	ND	ND	ND	4.72±0.64**	30
*sup-1*	4±0	4.02±0.14	9.80±1.01	0.72±0.54	NA	NA	50
*sup-1 ag-10*	4±0	4±0	>30^5^,**	NA	Indeterminate FM^6^ (100%)	NA	50
*sup-1 ag-10 pwr-1*	4±0	3.98±0.14	>30	NA	Indeterminate FM (100%)	NA	50
*sup-1 pwr-1*	4±0	4±0	10.80±1.73**	1.28±0.45**	NA	NA	50
*clv3-1*	ND	ND	ND	4.24±0.72	0%	3.32±0.68	50
*clv3-1 ag-10*	ND	ND	ND	4.20±1.07	0%	2.80±0.74**	50
*clv3-1 ag-10 pwr-1*	ND	ND	ND	6.23±1.01**	47%	1.35±0.36**	30
*crc-1*	4±0	4±0	5.86±0.35	2.02±0.14	0%	ND	50
*crc-1 pwr-1*	4±0	4±0	5.84±0.37	2.20±0.45**	76%	ND	50

Values indicate the average and standard deviation. T-tests were used to determine whether differences between genotypes were statistically significant. * and ** indicate p<0.05 and p<0.01, respectively, when comparing the data for a given genotype to the data in the same column for the genotype in the row immediately above it. Please note the following exception: *sup-1 pwr-1* data are compared with the data for *sup-1*.

1 The presence of internal organs was determined by dissecting gynoecia.

2 The ratio of gynoecium length to gynoecium width was used to quantify the bulged carpel phenotype; lower values correlate with more severe bulgedness.

3 NA indicates not applicable.

4 ND indicates that the value was not determined.

5 *sup-1 ag-10 pwr-1* flowers indeterminately produce stamen; “>30” indicates that all of the flowers analyzed (100%) had more than 30 stamens.

6 Indeterminate FM indicates a visible mass of undifferentiated cells in the center of the flower.

In addition to the rescue analysis, a T-DNA insertion line in the Columbia (Col) background, SALK_071811C (hereafter referred to as *pwr-2*), was obtained from the Arabidopsis Biological Resource Center [26]. In this mutant, the T-DNA insertion is near the 3′ end of the second exon of *PWR* (Figure 1A). To determine whether *pwr-2* also affects floral determinacy, *pwr-2* was crossed to *ag-10^Col^*, a line in which the *ag-10* mutation was backcrossed into Col six times. Unlike *ag-10* in the Landsberg *erecta* (L*er*) background, *ag-10^Col^* siliques are nearly indistinguishable from wild-type siliques, indicating the presence of a genetic suppressor of determinacy defects in Col [3]. *ag-10^Col^ pwr-2* double-mutant flowers were found to exhibit weak determinacy defects, namely, the infrequent production of visibly bulged gynoecia containing additional floral organs inside (Figure 1I). These findings and the *ag-10 pwr-1* double mutant phenotype indicate that *PWR* contributes to floral stem cell termination.

### *PWR* impacts other developmental processes

**Figure 2:**
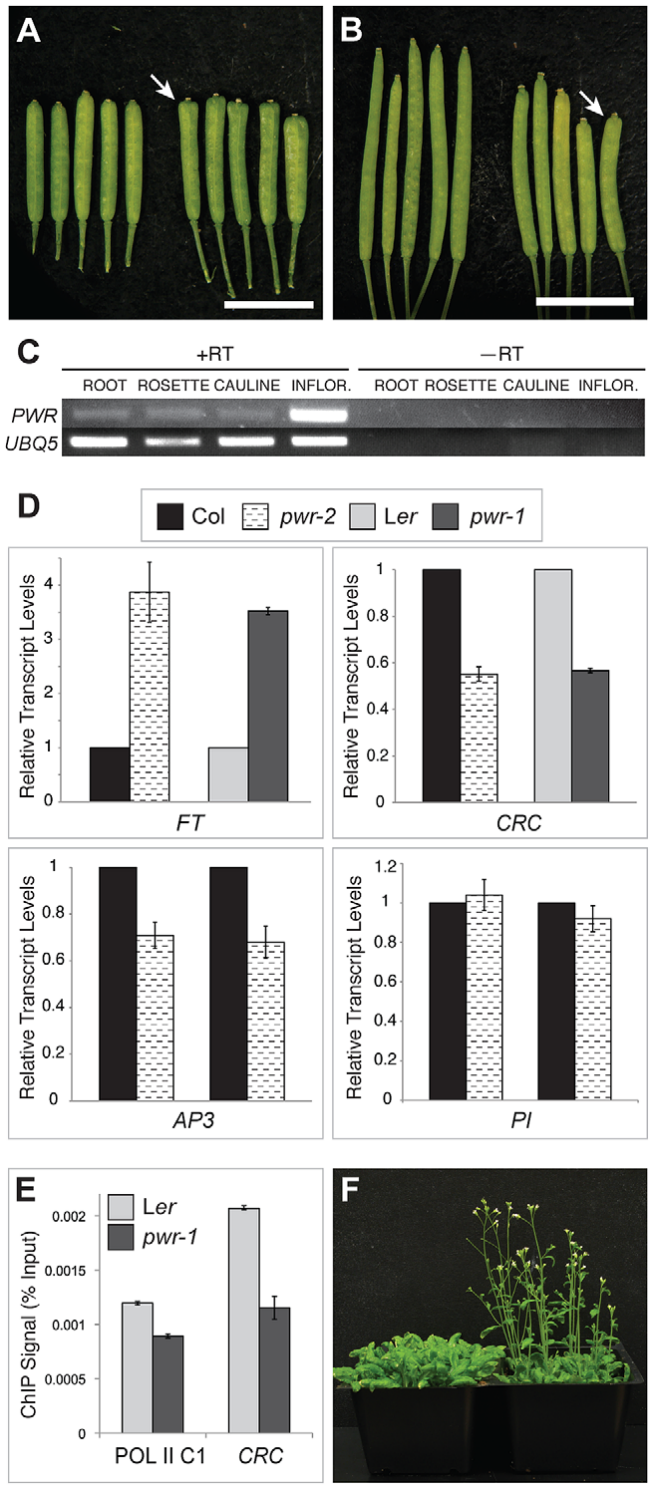
Phenotypes of *pwr-1* and *pwr-2* and transcript levels of genes involved in floral development. (A) L*er* (left) and *pwr-1* (right) siliques. (B) Col (left) and *pwr-2* (right) siliques. In (A) and (B), arrows indicate the bulged carpel tips of *pwr-1* and *pwr-2* siliques, and scale bars = 5 mm. (C) RT-PCR for *PWR* using cDNA obtained from L*er* roots, rosette leaves, cauline leaves, and inflorescences. *UBIQUITIN 5* was used as the control. The highest abundance of the *PWR* transcript was observed in inflorescences. (D) Real-time RT-PCR analysis of the transcript levels of *AP3*, *PI*, *CRC*, and *FT*. For *AP3* and *PI*, two biological replicates are shown for *pwr-2* compared to Col. For *CRC* and *FT*, data is shown for both *pwr* alleles. For all genes tested, transcript levels were normalized to *UBIQUITIN 5* and compared to the respective wild-type control. Error bars indicate the standard deviation for triplicate technical replicates. (E) Pol II occupancy at the *CRC* promoter determined by ChIP using anti-RPB2 antibodies in L*er* and *pwr-1*. Pol II C1, located in the intergenic region between At2g17470 and At2g17460, has no appreciable Pol II occupancy as determined in a previous study [51] and is used as a negative control. (F) Col (left) and *pwr-2* (right) plants grown side by side under 24-hour light conditions.

Both *ag-10 pwr-1* and *ag-10^Col^ pwr-2* mutants exhibited pleiotropic defects in addition to those in floral determinacy, including reduced plant height, early flowering, and aberrant petal shape. Although the defects were generally more pronounced in the L*er* ecotype (e.g., *ag-10 pwr-1* petals [Figure 1D] versus *ag-10^Col^ pwr-2* petals [Figure 1G]), pleiotropic phenotypes were observed in both the *pwr-1* and *pwr-2* single mutants. Defects in floral determinacy were not observed in the single mutants, but carpel development was visibly affected in both *pwr* alleles. *pwr-1* and *pwr-2* siliques were slightly flattened with the carpels bulged at the tip surrounding the stigmatic tissue (Figure 2A, 2B; the name *POWERDRESS* refers to the resemblance of the bulged carpel tips of the single mutants to the excessively padded shoulders of suit jackets from the 1980s).

RT-PCR analysis for *PWR* in L*er* wild-type roots, rosette leaves, cauline leaves, and inflorescence tissues revealed high *PWR* transcript abundance in inflorescences (Figure 2C). To help establish a general idea of how *PWR* might function in various developmental pathways, microarray analysis was performed using *pwr-2* and Col inflorescence tissue. Increased transcript levels of several genes with related functions in the specification of the floral meristem, including *FLOWERING LOCUS T* (*FT*), *SUPPRESSOR OF CONSTANS 1* (*SOC1*), *CAULIFLOWER* (*CAL*), and *SEPALLATA4* (*SEP4*), were observed in *pwr-2* (fold-change ≥1.5, p-value≤0.005) (Table S2). *FT* acts upstream of *SOC1*, and both integrate signals from various flowering time pathways to induce flowering by promoting the expression of floral meristem identity genes including *CAL* and *SEP4* [27]–[29]. Because these genes have closely related functions, it is likely that the transcript level changes observed for *SOC1*, *CAL*, and *SEP4* were indirect consequences of an increase in *FT* or another more upstream factor. The increase in *FT* was confirmed using real-time RT-PCR for both *pwr* alleles (approximately 3- to 4-fold increases, Figure 2D) and may be the underlying cause of the early flowering phenotype of the *pwr* mutants (Figure 2F).

Another related group of genes with altered transcript levels in *pwr-2* were three direct targets of the floral homeotic genes *APETALA3* (*AP3*) and *PISTILLATA* (*PI*): *BANQUO1* (*BNQ1*) and *BNQ2* and *NAC-LIKE*, *ACTIVATED BY AP3/PI* (*NAP*) [30], [31] (Table S1, Table S2). Although the changes in the transcript levels of all three genes suggested a decrease in *AP3* and/or *PI* function, neither of them was identified as having a significant change in expression in the microarray analysis. Real-time RT-PCR analysis for *PI* and *AP3* in *pwr-2* revealed no significant change in *PI* transcript levels and a small reduction for *AP3* (Figure 2D), which may explain the altered transcript levels of the *AP3*/*PI* target genes identified from the microarray analysis. However, the cause of the transcript level changes of *AP3/PI* targets remains unclear. Considering the role of *AP3* and *PI* in specifying the second and third whorl floral organs (petals and stamens, respectively), these transcript level changes may provide a starting point for investigating the petal defects observed in *pwr* mutants.

### *PWR* acts through *AP2*, *CRC*, and *WUS* in the floral determinacy network

To address whether *PWR* acts in any of the known pathways regulating floral determinacy, genetic analyses were carried out by combining *pwr-1* and *pwr-1 ag-10* with loss-of-function mutations in *CLAVATA3* (*CLV3*), *SUPERMAN* (*SUP*), *WUS*, *AG*, *CRC*, and *AP2*.

**Figure 3:**
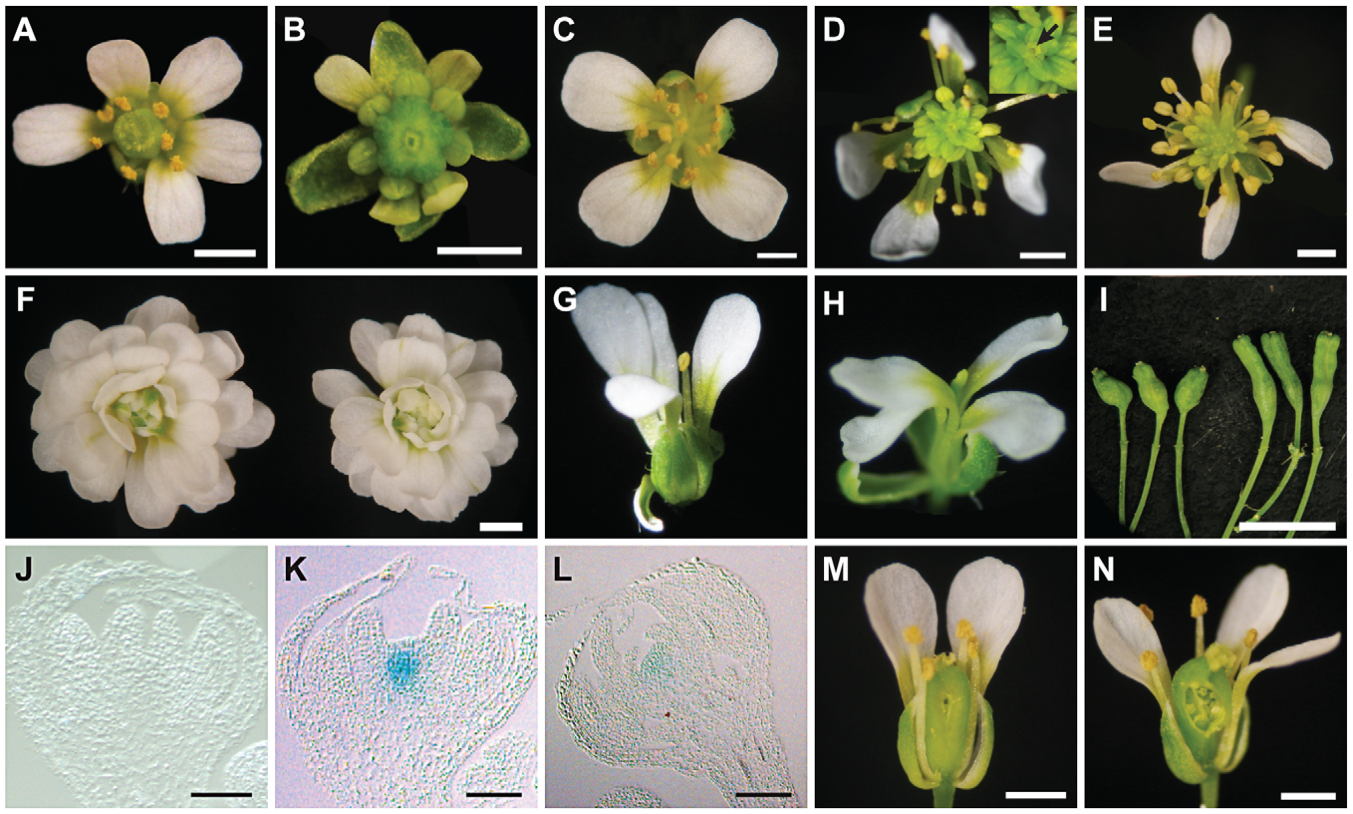
Phenotypes of *ag-10 pwr-1* in combination with loss-of-function mutations in other floral determinacy regulators. (A) *clv3-1* flower with supernumerary organs in all four whorls. (B) *clv3-1 ag-10 pwr-1* triple mutant flower. Compared to *clv3-1*, flowers of the triple mutant are small, have more carpels, and have shortened and bulged gynoecia often containing internal flowers. (C) *sup-1* flower with aberrant carpels and more stamens than wild-type. (D) *sup-1 ag-10* double mutant flower. These flowers are more strongly indeterminate compared to *sup-1*: more stamens are produced, and a mass of undifferentiated cells is visible at the center of the flower (magnified and indicated with an arrow in the inset). (E) *sup-1 ag-10 pwr-1* triple mutant flower. (F) *ag-1* (left) and *ag-1 pwr-1* (right) flowers. *pwr-1* does not enhance the indeterminacy defects of *ag-1* flowers. (G) *wus-1* flower exhibiting premature termination of floral development. (H) *wus-1 ag-10 pwr-1* flower. Along with *wus-1 pwr-1* (not shown), flowers of the triple mutant resemble *wus-1* single mutant flowers. (I) *ag-10 pwr-1* (left) and *ap2-2 ag-10 pwr-1* (right) gynoecia. The *ap2-2* mutation reduced the indeterminacy defects of *ag-10 pwr-1*: the triple mutant gynoecia were less bulged compared to *ag-10 pwr-1*, and a smaller percentage contained internal floral organs. (J) Longitudinal section of a stage 7 *ag-10 pWUS:GUS* flower showing the absence of GUS staining in the floral meristem. (K) Longitudinal section of a stage 7 or stage 8 *ag-10 pwr-1 pWUS:GUS* flower showing GUS staining in the floral meristem. (L) Longitudinal section of a stage 11 *ag-10 pwr-1 pWUS:GUS* flower. (M) *crc-1* flower and (N) *crc-1 pwr-1* flower with floral organs partially removed to reveal the gynoecia. While *crc-1* single mutant flowers have unfused carpels and rarely exhibit indeterminacy defects, approximately half of all *crc-1 pwr-1* gynoecia dissected contained internal floral organs. Scale bars = 10 µm in (J) to (L), 5 mm in (I), and 1 mm in all other panels.

Enhanced determinacy defects were found when *clv3-1* was combined with *ag-10 pwr-1*. *clv3-1* single mutant flowers produce slightly larger numbers of each floral organ type [32] including carpels (Figure 3A, Table 1). *clv3-1 ag-10* flowers resembled *clv3-1* flowers (Table 1). *clv3-1 ag-10 pwr-1* triple mutant flowers were more indeterminate in several respects (Figure 3B). First, *clv3-1 ag-10 pwr-1* produced a significantly larger number of carpels than *clv3-1* and *clv3-1 ag-10* (Table 1). Second, 47% of all triple mutant gynoecia that were dissected contained internal floral organs, which were never observed in *clv3-1* or *clv3-1 ag-10* (Table 1). The enhanced determinacy defects observed when *ag-10 pwr-1* was combined with *clv3-1* indicate that *PWR* confers determinacy in a pathway parallel to *CLV3*.

*sup-1* mutant flowers produce supernumerary stamens, and the increased number of floral organs produced indicates a role for *SUP* as a positive regulator of floral determinacy [33]. *sup-1 pwr-1* flowers had a small but statistically significant increase in organ number in the inner two whorls relative to *sup-1* flowers (Table 1). *ag-10* significantly enhanced the determinacy defects of *sup-1* (Table 1): *sup-1 ag-10* plants produced flowers with a significantly increased number of stamens and a visible group of undifferentiated cells in the center of the floral meristem (Figure 3D, Table 1). The *sup-1 ag-10 pwr-1* triple mutant produced flowers similar to those of the *sup-1 ag-10* double mutant (Figure 3E, Table 1). The lack of strong additive effects between *pwr-1* and *sup-1* suggests that *PWR* and *SUP* act in a common pathway in floral determinacy. However, how *SUP* impacts floral determinacy at the molecular level is unknown.

When crossed to *ag-1*, *pwr-1* failed to enhance the determinacy defects of the null *ag* allele, based on flower size and the number of petals produced in *ag-1 pwr-1* compared to *ag-1* (Figure 3F). This suggested that *PWR* largely acted through the *AG* pathway to confer floral determinacy. Since *AG* is required for shutting off *WUS* expression in floral meristems at stage 6, further analysis was performed to assess whether *PWR* acted upstream of *WUS* in the regulation of floral determinacy. *wus-1 pwr-1* and *wus-1 ag-10 pwr-1* plants produced prematurely terminated *wus-1*-like flowers and failed to form the innermost floral organs (Figure 3G, 3H, *wus-1 pwr-1* not shown). In addition to this analysis, crosses to a *pWUS:GUS* reporter line (for β-glucuronidase) containing a 3.2-kb *WUS* promoter [3], [34] were performed. In wild-type floral meristems, this reporter is active between floral stages 1 and 6 [3]. In the *ag-10* background, *GUS* expression was only observed in approximately 10% of flowers beyond stage 6 (Figure 3J) [3], [34]. In *ag-10 pwr-1*, 90% of flowers (nine out of ten) showed prolonged *GUS* expression in late stage floral meristems (Figure 3K, 3L), indicating that *WUS* expression was prolonged in *ag-10 pwr-1*. Taken together, these analyses indicate that *PWR* confers determinacy in the same pathway as *AG* and that *PWR* acts upstream of *WUS*.

The YABBY transcription factor *CRC* has repeatedly been shown to promote floral determinacy, most demonstrably when *crc-1* is combined with mutations in other floral determinacy genes [4]–[6]. The microarray analysis in *pwr-2* indicated that *CRC* transcript levels were reduced in the mutant, and the ∼2-fold reduction was confirmed in *pwr-1* and *pwr-2* by real-time RT-PCR analysis (Table S1, Figure 2D). The relationship between *PWR* and *CRC* with respect to floral determinacy was further assessed by generating the *crc-1 pwr-1* double mutant. Although neither *crc-1* nor *pwr-1* produced internal floral organs within the gynoecium, internal floral organs were observed in 76% of the *crc-1 pwr-1* gynoecia dissected (Figure 3M, 3N, Table 1). This double mutant phenotype together with the reduced *CRC* transcript levels in both *pwr* mutants raises the possibility that *PWR* promotes floral determinacy through *CRC* and through a pathway parallel to *CRC*.

In contrast to *AG*, *SUP*, and *CRC*, which promote determinacy, *AP2* promotes stem cell maintenance. *AP2* has an antagonistic or complementary relationship with *AG* in the flower [8], [21]. Its role in floral stem cell maintenance has been evidenced by the severely indeterminate flowers produced when *AP2* is simultaneously misexpressed and resistant to regulation by miR172 [8]. Compared to *ag-10 pwr-1*, *ap2-2 ag-10 pwr-1* triple mutant plants exhibited a less severe indeterminate phenotype, indicated by reduced bulging and a reduced percentage of flowers containing internal floral organs (Figure 3I, Table 1). This suggested that the floral determinacy defects of *ag-10 pwr-1* were partially due to increased *AP2* expression or activity.

### *PWR* promotes the expression of *CRC* and several *MIR172* genes at the transcriptional level

**Figure 4:**
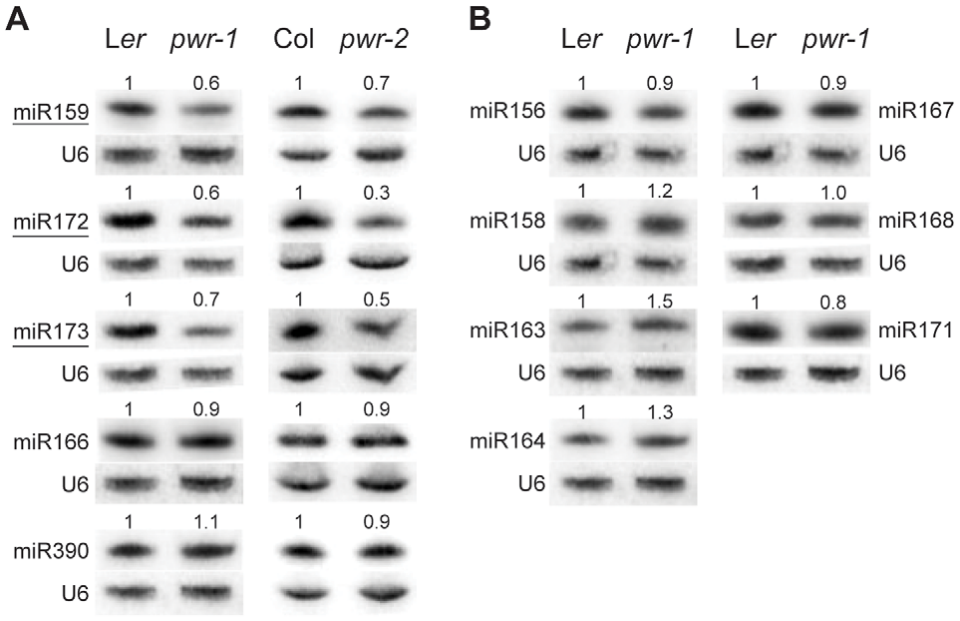
miRNA abundance in *pwr-1* and *pwr-2*. (A) Abundance of miR159, miR172, miR173, miR166, and miR390 in L*er*, *pwr-1*, Col, and *pwr-2* detected by small RNA northern blotting. The abundance of miR159, miR172, and miR173 (underlined) was reduced in both *pwr* alleles relative to their respective controls. Decreased accumulation was not observed for miR166 or miR390 in either allele. (B) Additional miRNAs tested in *pwr-1* did not have reduced abundance compared to L*er*. In (A) and (B), values indicate the relative abundance of the indicated miRNA species in *pwr-1* compared to L*er* or in *pwr-2* compared to Col. For all blots, ImageJ signal intensity analysis was used for quantification. The numbers above the miRNA blots indicate the relative miRNA abundance between the mutants and wild type.

Because *AP2* is under the regulation of miR172 in the flower, experiments were performed to assess whether miR172 accumulation or activity was compromised by mutations in *PWR*. Small RNA northern blotting analysis was performed to measure the levels of miR172 in *pwr-1* and *pwr-2*. The relative levels of miR172 in *pwr-1* and *pwr-2* were 0.6 and 0.3, respectively, compared to their corresponding wild-type controls (Figure 4A). Next, the levels of *AP2* mRNA and AP2 protein in inflorescences were determined using real-time RT-PCR and western blotting. Differences in *AP2* mRNA and protein levels were not observed between *pwr-1* and L*er* or between *ag-10 pwr-1* and *ag-10*. This was not surprising because the expression of *AP2* in the outer two whorls may have masked the effects of miR172 in the small number of floral stem cells. Utilizing a different approach, the 3′ cleavage products from the *AP2* mRNA were detected to directly evaluate the activity of miR172. The levels of the 3′ cleavage products were reduced in *pwr-1* relative to L*er* and in *ag-10 pwr-1* relative to *ag-10* (Figure S1). Therefore, the reduced miR172 levels in *pwr* likely compromised the full repression of *AP2* by miR172.

**Figure 5:**
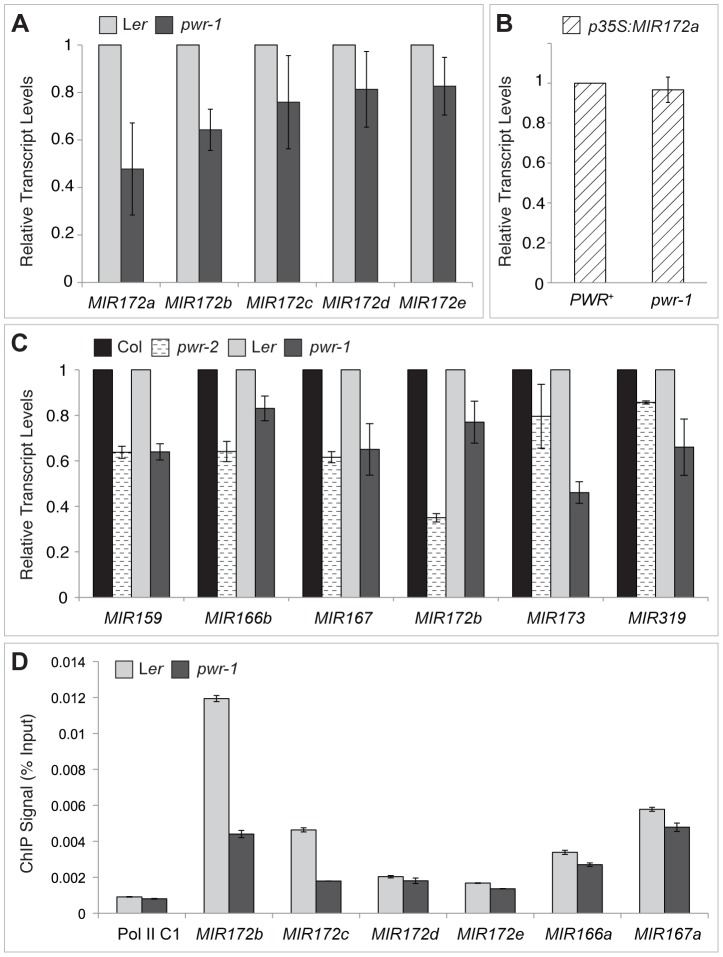
pri–miRNA abundance and Pol II occupancy at *MIR* genes in *pwr-1* and *pwr-2*. (A) Transcript levels of pri-miR172a-e in *pwr-1*. pri-miR172a and pri-miR172b transcript levels were reduced in *pwr-1*, while no significant changes were observed for pri-miR172c, pri-miR172d, and pri-miR172e. (B) Transcript levels of pri-miR172a in *PWR^+^* (*PWR*/*PWR* and *PWR*/*pwr-1*) and *pwr-1* plants harboring a *p35S:MIR172a* transgene. (C) Real-time RT-PCR analysis of other pri-miRNAs. A general decrease was observed in pri-miRNA transcript levels in both *pwr* alleles. For all pri-miRNAs tested, transcript levels were normalized to *UBIQUITIN 5* and compared to the respective wild-type control. In (A) to (C), error bars indicate the standard deviation for triplicate technical replicates. Three biological replicates gave similar results. (D) Pol II occupancy at *MIR* loci determined by ChIP using anti-RPB2 antibodies in L*er* and *pwr-1*. Error bars indicate the standard deviation for triplicate technical replicates. Pol II C1, located in the intergenic region between At2g17470 and At2g17460, has no appreciable Pol II occupancy as determined in a previous study [51] and is used as a negative control.

The next question addressed was how *PWR* promotes the accumulation of miR172. Several lines of evidence suggested it was unlikely that miRNA biogenesis genes were compromised in the *pwr* mutants. First, northern blot analysis revealed that reductions in mature miRNA abundance were restricted to three (miR172, miR173, and miR159) of the examined miRNA species in both *pwr* alleles (Figure 4). Second, the expression levels of known miRNA biogenesis genes were not affected in *pwr-2* according to the results from the microarray analysis. Additionally, there were no differences in pri-miR172a levels in *PWR^+^* (both *PWR/PWR* and *PWR/pwr*) and *pwr-1* plants harboring a *p35S:MIR172a* transgene (Figure 5B). Taken together, these findings suggested that the involvement of *PWR* in miRNA biogenesis is not downstream of transcription. To determine if *PWR* affected the transcription of individual *MIR172* genes, real-time RT-PCR was performed for all five miR172 pri-miRNAs in *pwr-1*. Decreased transcript levels of pri-miR172a and pri-miR172b were observed in *pwr-1*, while the three remaining pri-miRNAs exhibited only subtle changes or were not affected (Figure 5A).

To address the possibility that *PWR* might affect the transcription of other *MIR* genes, the abundance of other pri-miRNAs were determined using real-time RT-PCR. In both *pwr-1* and *pwr-2*, compared to their respective wild-type controls, there was a general reduction in the transcript levels of the pri-miRNAs tested (pri-miR159, pri-miR166b, pri-miR167, pri-miR173, and pri-miR319) (Figure 5C). The occupancy of Pol II at the promoters of several miRNA genes and *CRC* was examined by chromatin immunoprecipitation (ChIP). Pol II occupancy was clearly reduced at the *MIR172b*, *MIR172c*, and *CRC* loci in *pwr-1* (Figure 5D, Figure 2E), while only mild changes or no changes were observed at the *MIR172d*, *MIR172e*, *MIR166a*, and *MIR167a* loci (Figure 5D). These findings indicate that *PWR* specifically regulates *CRC* and some, but not all, of the *MIR172* genes through the recruitment of Pol II to these loci.

### *MIR172d* is required for floral determinacy

**Figure 6:**
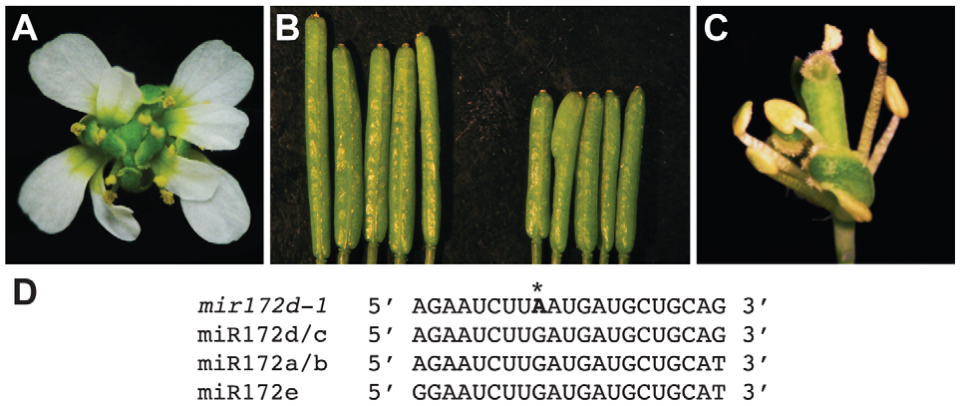
Phenotype of the *mir172d-1* single mutant and of *mir172d-1* in combination with *ag-10* and *ap2-2*. (A) *ag-10 mir172d-1* flower. (B) L*er* (left) and *mir172d-1* (right) siliques. The gynoecia of *mir172d-1* occasionally have three fused carpels instead of two. (C) *ap2-2 ag-10 mir172d-1* flower. (D) Mature miR172 sequences and the site of the G-to-A mutation in *mir172d-1* (indicated by *).

Coincidental with the observation that miR172 levels were reduced in *pwr* mutants, the mutation in another enhancer isolated from the *ag-10* genetic screen was mapped to the *MIR172d* locus. A G-to-A mutation was identified at the ninth position of the 21-nucleotide sequence that corresponds to the mature miRNA (Figure 6D), and the mutation was designated *mir172d-1*. The enhanced floral determinacy phenotype of *ag-10 mir172d-1* ranged from bulged gynoecia to the flower-within-flower phenotype, in which the fourth whorl organs were replaced with another flower (Figure 6A). The gynoecia of *mir172d-1* single mutant flowers were not bulged but were shorter than those of L*er* and occasionally consisted of three fused carpels instead of two (Figure 6B).

To verify that *MIR172d* confers floral determinacy through the *AP2* pathway, the status of miR172-mediated cleavage of *AP2* mRNA was first evaluated in *ag-10* and *ag-10 mir172d-1* inflorescences. Semi-quantitative 5′ RACE PCR showed that the 3′ cleavage products from *AP2* mRNA were reduced in *ag-10 mir172d-1* relative to *ag-10* (Figure S1), suggesting that miR172-mediated repression of *AP2* was compromised. In addition, the *ag-10 ap2-2 mir172d-1* triple mutant was generated to determine whether the floral determinacy defects of *ag-10 mir172d-1* could be suppressed by *ap2-2*. The phenotype of the triple mutant clearly demonstrates that miR172-mediated repression of *AP2* is required for floral determinacy as the *ap2-2* mutation completely rescued the floral determinacy defects of *ag-10 mir172d-1* (Figure 6C). Neither the flower-within-flower phenotype nor bulged carpels were observed in the triple mutant. Although a role for miR172 as a positive regulator of floral determinacy has previously been inferred from findings that established it as a negative regulator of *AP2* in the flower, the *ag-10 mir172d-1* phenotype provides direct loss-of-function evidence that at least one of the *MIR172* genes, *MIR172d*, is required for the proper termination of the floral stem cells.

miR172 is also known to promote flowering by targeting several members of the *AP2* gene family in addition to *AP2* [18]. Interestingly, the *mir172d-1* mutation did not affect flowering time (data not shown), suggesting that other *MIR172* genes are sufficient to confer this developmental function.

## Discussion

### *PWR* contributes to the robustness of the floral determinacy network through *MIR172* and *CRC*

**Figure 7:**
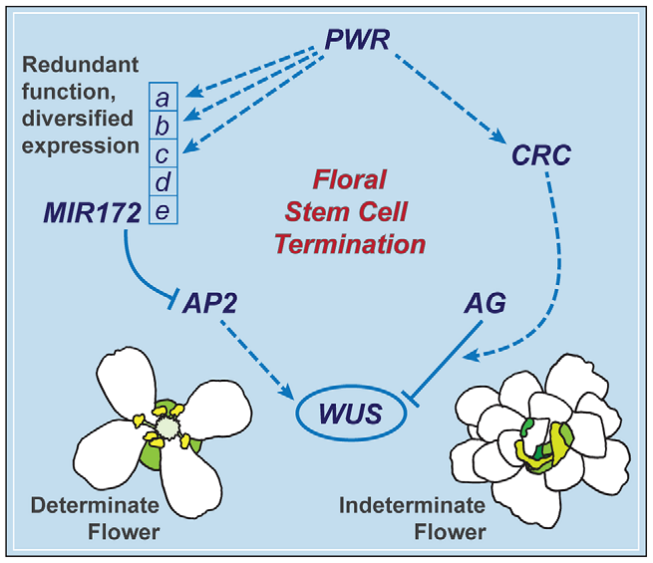
A summary of the floral determinacy gene network highlighting the function of *PWR*. *AP2* and *AG* act antagonistically in terms of the regulation of *WUS* expression: *AP2* promotes *WUS* expression and stem cell maintenance, while *AG* represses *WUS* expression to elicit stem cell termination. *CRC* acts downstream of *AG*, and miR172 represses *AP2* expression. *PWR* promotes floral stem cell termination by enhancing the expression of *CRC* and three of the five *MIR172* genes. *MIR172d*, whose expression is independent of *PWR*, is functional in the repression of *AP2* expression and the control of floral determinacy.

The termination of the floral meristem requires various regulatory factors whose functions are orchestrated during the different stages of floral development. The data presented here indicate that a previously uncharacterized gene, *PWR*, confers floral determinacy through two distinct players: miR172 and *CRC* (Figure 7), which are active in the flower at different developmental stages. miR172 is present in the inner whorls from early stages onward [9], [21], while *CRC* is not expressed until carpel growth is initiated in late-stage flowers [35]. In addition to the possibility that the effect of *PWR* on floral stem cell termination is temporally distributed, the changes in miR172 accumulation and pri-miR172 transcript levels in *pwr-1* shed light on how *PWR* contributes to the robustness of the floral determinacy network. The impact of *PWR* on multiple but not all of the five *MIR172* genes and the finding that a mutation in *MIR172d*, whose expression is independent of *PWR* (Figure 7), also affects determinacy demonstrate that the expression diversification of this miRNA family is functionally relevant. Diversification in the regulation of the *MIR172* genes and their common function in negatively regulating *AP2* make the stem cell termination network more robust. This is reflected by the fact that loss of function in *PWR* (and consequently reduced expression of *MIR172a-c*) or *MIR172d* each has little effect on proper floral determinacy in the wild-type background.

### *PWR* contributes to other floral developmental pathways

Although determinacy defects were not observed in single *pwr* mutants, the other pleiotropic defects initially observed in *pwr-1 ag-10*, including early flowering, petal defects, and small plant size, were observed in *pwr-1*. Some preliminary hypotheses can be drawn from the microarray and transcript level analysis, including the possible role of increased *FT* expression and decreased *AP3* expression in disrupted flowering time and petal development, respectively. Although further investigation of the pleiotropic defects of *pwr* is required, the data clearly indicate that the defects are not attributable to widespread changes in miRNA abundance, as is the case in miRNA biogenesis mutants. First, the early flowering phenotype of *pwr* is in contrast to the late-flowering phenotype expected for a reduction of miR172 and is instead consistent with the increased *FT* transcript levels observed. Second, the widespread reduction observed for pri-miRNA levels in *pwr* were not accompanied by widespread decreases in mature miRNA abundance. One possible explanation for this discrepancy is that decreases in precursor levels do not always correspond to decreases in mature miRNAs, due in part to differences in the post-transcriptional maturation mechanisms for individual miRNAs [36], [37].

The predominant feature of the predicted PWR protein is a pair of SANT domains encoded in the fourth and largest exon of the gene. A BLAST search for proteins similar to PWR in *Arabidopsis* and other organisms identified PWR orthologs in other plant species but no homologs in *Arabidopsis*. Furthermore, PWR was found to have higher amino acid sequence similarity to histone deacetylase (HDAC) complex proteins in animals than to any other proteins in *Arabidopsis*. Studies in animals and yeast have uncovered the involvement of SANT domain-containing enzyme subunits in chromatin remodeling activity, including histone acetylation, histone deacetylation, and ATP-dependent chromatin remodeling [25]. The presence of two SANT domains in *PWR* and the spacing between them (173 amino acids) are similar to the domain structures of the HDAC subunits SMRT and N-CoR (nuclear receptor co-repressors), whose first and second SANT domains are necessary for HDAC activation and binding to unacetylated histone tails, respectively [38], [39]. Although further studies are necessary to determine whether PWR acts as a chromatin remodeling factor, this potential mode of activity may help explain how altered *PWR* function yields pleiotropic defects and changes in the transcript levels of otherwise unrelated genes, including *MIR172B*, *CRC*, and *FT*.

### Individual *MIR172* genes may have distinct developmental functions

In addition to its role in floral development, miR172 regulates flowering time as a negative regulator of floral repressors that belong to the AP2 transcription factor family [18]. The first characterization of miR172 function was made possible by transgenic lines in which miR172 was overexpressed (yielding early-flowering and *ap2*-like phenotypes) or lines in which the miR172 target *AP2* was made resistant to regulation by the miRNA [9], [40]. Although the latter effectively corresponds to loss of miR172-mediated repression of *AP2*, it does not distinguish among the mature miR172 produced from the five *MIR172* loci. Previous reports of differences in the accumulation of the individual pri-miRNAs have suggested variations in their individual functions throughout the plant. For example, the pri-miR172b precursor is particularly abundant in the shoot apex at the induction of flowering [41]. The *miR172d-1* mutation provides direct genetic evidence for the role of miR172 as a positive regulator of floral determinacy, and the absence of flowering time defects in this mutant suggests that the developmental functions (flowering time and floral determinacy) assigned to miR172 can be uncoupled. Considered together with the effects of *PWR* on the transcription of some, but not all, of the *MIR172* genes, these findings also demonstrate that the contributions of individual members of a miRNA family to one particular developmental process can be distinguished.

## Materials and Methods

### Plant materials and growth conditions

The mutants and transgenic lines used in this study are in the Landsberg *erecta* (L*er*) ecotypic background, except for *pwr-2*, which is in the Columbia (Col) background, and *ag-10^Col^*, which is the *ag-10* mutation introgressed into Col through six consecutive backcrosses. All plants were grown at 23°C under long-day conditions (16 hours light, 8 hours dark). *ag-1* [23], *clv3-1* [32], *sup-1* [33], *ap2-2* [42], *wus-1* [43], *crc-1* [4], and *ag-10* [22] are previously described mutations.

*pwr-1* and *miR172d-1* were backcrossed into the *ag-10* background and L*er* two times prior to further analysis to reduce the number of extraneous background mutations. *pwr-2* is a T-DNA insertion line, SALK_0713811C, obtained from the Arabidopsis Biological Resource Center [26].

### Mapping of the *pwr-1* and *mir172d-1* mutations

The *ag-10 pwr-1 and ag-10 mir172d-1* mutants were isolated from the *ag-10* screen and crossed to *ag-10^Col^* to generate the respective mapping populations. For *ag-10 pwr-1*, 20 F2 plants exhibiting the enhanced phenotype were used for rough mapping, and linkage was observed for the ciw4 marker on the lower arm of chromosome 3. The mapping population was expanded to 888 plants for fine mapping. SSLP and CAPS markers were designed based on identified polymorphisms between the L*er* and Col ecotypes (http://arabidopsis.org/browse/Cereon/index.jsp). The mapping region was narrowed to a 154-kb window encompassing portions of two BAC clones, F4F15 and T25B15, and spanning 55 open reading frames. A G-to-A mutation that produces a premature stop codon in At3g52250*/PWR* was identified by sequencing analysis. For *ag-10 mir172d-1*, 24 F2 plants exhibiting the enhanced phenotype were used for rough mapping, and linkage was again observed for the ciw4 marker on chromosome 3. Using an expanded mapping population of approximately 200 plants, the mapping region was narrowed to an 1,100-kb window spanning portions of the BAC clones T26I12 and T16L24. The sequencing analysis revealed a G-to-A mutation in At3g55512*/MIR172d*.

### Genotyping

The primer sequences used for genotyping are listed in Table S3. For *pwr-1*, the dCAPS primers PWRgeno-F and PWRgeno-R produce an *Hph*I (NEB, Cat# R0158) site exclusively in the mutant. The *pwr-2* mutation was genotyped using primers EN1_XcmIF and 3G52250R1, which fail to amplify a fragment in the homozygous mutant. The *ag-10* mutation eliminates an *HpyA*V restriction site and was genotyped using primers AGp1 and ag10_genoR followed by *HpyA*V digestion (NEB, Cat# R0621). For *miR172d-1*, the primers R194geno-F and R194geno-R were used to amplify the genomic fragment. The fragment amplified from *miR172d-1* fails to be cut by *Hpy188*III (NEB, Cat# R0622).

### Plasmid construction

To generate *pPWR:PWR-GFP*, the *PWR* genomic region was amplified using primers EN1_full_CACC and EN1cDNA_NS (Table S3). The 8.1-kb genomic region includes 1,670 bp of the upstream promoter region and the entire coding region of *PWR*, excluding the stop codon and three preceding nucleotides. The fragment was cloned into pENTR/D-TOPO (Invitrogen, Cat# K2400-20) and introduced into the pMDC107 Gateway-compatible vector [44]. *ag-10 pwr-1* and *pwr-1* plants were transformed with the plasmid by agroinfiltration for the phenotypic rescue analysis.

### RNA extraction and real-time RT–PCR

The harvesting of inflorescence tissue was consistently performed at the same time of day. RNA was extracted using TRI reagent (MRC, Cat# TR118), and DNA was removed using DNase I (Roche, Cat# 04716728001). Oligo-dT primer (Fermentas, Cat# S0131) and reverse transcriptase (Fermentas, Cat# EP0441) were used to synthesize cDNA. All procedures used were according to the manufacturers' instructions. Quantitative PCR was performed in triplicate on a Bio-Rad IQ cycler apparatus using iQ SYBR Green Supermix (Bio-Rad, Cat#170-0082). The primers used for real-time RT-PCR were previously described or designed using the Primer3 online tool (http://biotools.umassmed.edu/bioapps/primer3_www.cgi) and are listed in Table S3.

### Small RNA Northern blot analysis

The hybridization and detection of miRNAs were performed as previously described [45]. Five micrograms total RNA was obtained from inflorescence tissues, and 5′-end-labeled ^32^P antisense DNA oligonucleotides were used to detect mature miRNA species. U6 was used as the loading control. The oligonucleotide probes used were previously described [46], [47] and are listed in Table S3. The signal intensities of the blots were quantified using ImageJ processing and analysis software (http://rsbweb.nih.gov/ij/).

### ATH1 Affymetrix microarray analysis

Total RNA extracted from inflorescences was purified using the RNeasy Plant Mini Kit (Qiagen, Cat# 74903). Affymetrix Gene Chip probe preparation, hybridization, and quality control were performed according to the manufacturer's instructions. The SimpleAffy package available for the R language was used to analyze the microarray data, and expression values were determined using the MAS 5.0 method as previously described [48]. Changes in gene expression were defined as transcript level fold-changes ≥1.5 with p-values≤0.005.

### Histochemical staining

GUS staining was performed as previously described [49], [50]. Inflorescences were fixed in 90% cold acetone for 15 to 20 min and rinsed with the rinse solution [50 mM NaPO_4_, pH 7.2; 0.5 mM K_3_Fe(CN)_6_; and 0.5 mM K_4_Fe(CN)_6_]. After the infiltration solution [50 mM NaPO_4_, pH 7.2; 0.5 mM K_3_Fe(CN)_6_; 0.5 mM K_4_Fe(CN)_6_; and 2 mM X-Gluc] was added, the inflorescences were vacuum infiltrated for 10 min then incubated at 37°C overnight.

### Chromatin immunoprecipitation

ChIP was performed as previously described [3]. Inflorescences were ground in liquid nitrogen and cross-linked with 1% formaldehyde in M1 buffer [10 mM phosphate buffer, pH 7.0; 0.1 M NaCl; 10 mM mercaptoethanol; 1 M hexylene glycol, 1× protease inhibitor cocktail (Roche); and 1 mM PMSF] for 10 min. The suspension was filtered through four layers of Miracloth, and the filtrate was centrifuged at 12,000 rpm for 10 min. The pelleted chromatin was washed three times with M2 buffer (M1 buffer plus 10 mM MgCl_2_ and 0.5% Triton X-100) and one time with M3 buffer [10 mM phosphate buffer, pH 7.0; 0.1 M NaCl; 10 mM mercaptoethanol; 1× protease inhibitor cocktail (Roche); and 1 mM PMSF]. The chromatin was resuspended in nuclei lysis buffer and sonicated to generate DNA fragments approximately 500 bp in length. The lysate was pre-cleared by incubation with 50 µL protein-A agarose beads/salmon sperm DNA (Millipore) for 1 h then incubated with anti-RBP2 antibody (Abcam 10338) overnight. The bound DNA fragments were recovered and purified using columns from the Qiagen Plasmid Extraction Kit according to the manufacturer's instructions. Quantitative real-time PCR was performed on bound and input DNAs. The primers used are listed in Table S3.

### 5′ RACE to detect 3′ cleavage products from *AP2* mRNA

Total RNA was extracted from dissected inflorescences, and mRNA was isolated using the Sera-Mag Magnetic Oligo(dT) kit (Thermo Scientific) according to the manufacturer's instructions. 5′ RACE was performed using the GeneRacer Kit (Invitrogen) according to the manufacturer's instructions. Briefly, an RNA oligonucleotide adaptor was ligated to the isolated mRNAs, and reverse transcription was conducted using oligo-dT. *AP2* 3′ cleavage products were amplified and sequenced. For amplification, a primer specific to the 5′ adaptor and an *AP2*-specific primer were used.

## Supporting Information

Figure S1 *pwr-1* and *mir172d-1* reduce the accumulation of the 3′ cleavage products from *AP2* mRNA. The 3′ products resulting from cleavage of *AP2* mRNA by miR172 were detected using 5′ RACE RT-PCR for L*er*, *ag-10*, *pwr-1*, *ag-10 pwr-1*, and *ag-10 mir172d-1*. *UBIQUITIN 5* was used as the loading control. *ACTIN2* (*ACT2*) RT-PCR was conducted using an intron primer and an exon primer and served as a control for DNA contamination. The serial dilutions for L*er* cDNA show that the PCR results are semi-quantitative (“1” stands for undiluted samples).
(TIF)

Table S1 Genes down-regulated in *pwr-2*.
(PDF)

Table S2 Genes up-regulated in *pwr-2*.
(PDF)

Table S3 Sequences of oligonucleotide used in this study.
(PDF)
