# Supplementary figures and images for: POWERDRESS and Diversified Expression of the MIR172 Gene Family Bolster the Floral Stem Cell Network
Source: PLoS Genet. 2013 Jan 17;9(1):e1003218. doi: 10.1371/journal.pgen.1003218 (PMC3547843; doi:10.1371/journal.pgen.1003218)

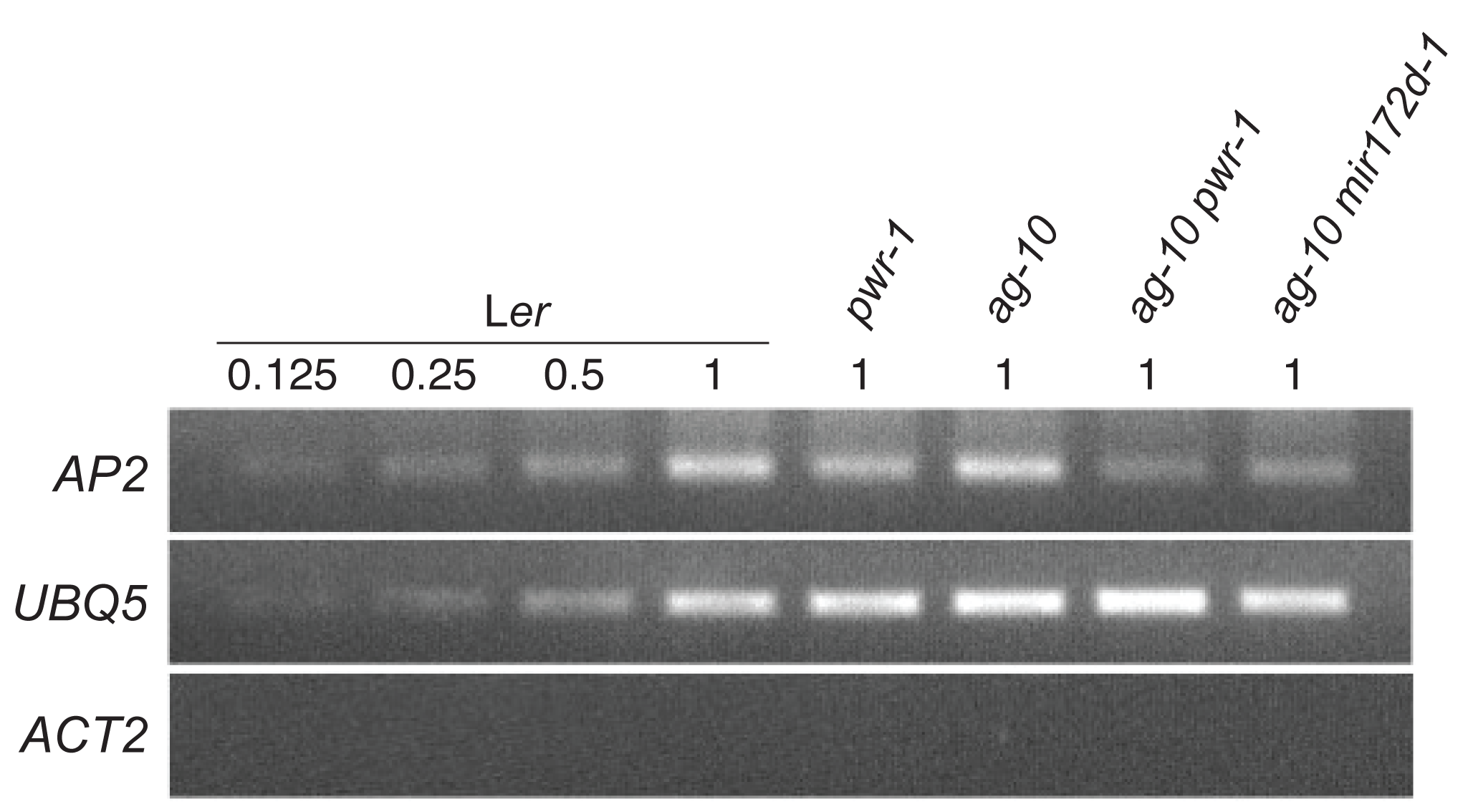

Supplement: Figure S1 — pwr-1 and mir172d-1 reduce the accumulation of the 3′ cleavage products from AP2 mRNA. The 3′ products resulting from cleavage of AP2 mRNA by miR172 were detected using 5′ RACE RT-PCR for Ler, ag-10, pwr-1, ag-10 pwr-1, and ag-10 mir172d-1. UBIQUITIN 5 was used as the loading control. ACTIN2 (ACT2) RT-PCR was conducted using an intron primer and an exon primer and served as a control for DNA contamination. The serial dilutions for Ler cDNA show that the PCR results are semi-quantitative (“1” stands for undiluted samples). (TIF) [file pgen.1003218.s001.tif]
