# Supplementary material for: POWERDRESS and Diversified Expression of the MIR172 Gene Family Bolster the Floral Stem Cell Network
Source: PLoS Genet. 2013 Jan 17;9(1):e1003218. doi: 10.1371/journal.pgen.1003218 (PMC3547843; doi:10.1371/journal.pgen.1003218)
Supplement: Table S1 — Genes down-regulated in pwr-2. (PDF) [file pgen.1003218.s002.pdf]

Table S1. Genes down-regulated in *pwr-2*. Highlighted rows indicate genes discussed in the text.

Genes down-regulated in *pwr-2* (Fold change ≥ 1.5, p-value ≤ 0.005)				
**Affymetrix code**	**Abundance (*pwr-2* / Col)**	**AGI code**	**Name(s)**	**Description**
253161_at	0.1601	At4g35770	DIN1, SEN1	Senescence-associated gene that is strongly induced by phosphate starvation.
256940_at	0.1639	At3g30720	QQS	QUA-QUINE STARCH (QQS)
266746_s_at	0.1791	At2g02930	GST16	Encodes glutathione transferase belonging to the phi class of GSTs
245265_at	0.2472	At4g14400	ACD6	encodes a novel protein with putative ankyrin and transmembrane regions
248062_at	0.2477	At5g55450		protease inhibitor/seed storage/lipid transfer protein (LTP) family protein
258225_at	0.2893	At3g15630		unknown protein
262119_s_at	0.3112	At1g02930	GST16	Encodes glutathione transferase belonging to the phi class of GSTs
258791_at	0.3296	At3g04720	HEL, PR4	Encodes a protein similar to the antifungal chitin-binding protein hevein from rubber tree latex
257315_at	0.3302	At3g30775	ERD5	Encodes a proline oxidase that is predicted to localize to the inner mitochondrial membrane
263431_at	0.3350	At2g22170		Lipase/lipooxygenase, PLAT/LH2 family protein
245038_at	0.3457	At2g26560	PLA, PLA2A, PLP2	encodes a lipid acyl hydrolase
263776_s_at	0.3640	At2g46440	CNGC11	Member of Cyclic nucleotide gated channel family
260556_at	0.3732	At2g43620		chitinase family protein
248092_at	0.3754	At5g55170	SUMO3	encodes a small ubiquitin-like modifier (SUMO) polypeptide
257334_at	0.3803	orf111d		
251065_at	0.3879	At5g01870		Predicted to encode a PR (pathogenesis-related) protein
267076_at	0.4287	At2g41090		calcium-binding EF-hand family protein
250669_at	0.4296	At5g06870	PGIP2	polygalacturonase inhibiting protein 2 (PGIP2)
262286_at	0.4316	At1g68585		unknown protein
251356_at	0.4333	At3g61060	AtPP2-A13	phloem protein 2-A13
266984_at	0.4482	At2g39570		ACT domain-containing protein
265117_at	0.4528	At1g62500		Bifunctional inhibitor/lipid-transfer protein/seed storage 2S albumin superfamily protein
244951_s_at	0.4608	ccb452		NA
262616_at	0.4688	At1g06620		similar to a 2-oxoglutarate-dependent dioxygenase
256965_at	0.4690	At3g13450	DIN4	branched chain alpha-keto acid dehydrogenase E1 beta
248435_at	0.4699	At5g51210	OLEO3	Encodes oleosin3, a protein found in oil bodies
250445_at	0.4744	At5g10760		aspartyl protease family protein
256300_at	0.4805	At1g69490	NAP	Encodes a member of the NAC transcription factor gene family. It is expressed in floral primordia and upregulated by AP3 and PI.
264355_at	0.4805	At1g03210		phenazine biosynthesis PhzC/PhzF family protein
253258_at	0.4854	At4g34400		AP2/B3-like transcriptional factor family protein
249073_at	0.4916	At5g44020		HAD superfamily, subfamily IIIB acid phosphatase

Genes down-regulated in *pwr-2* (Fold change ≥ 1.5, p-value ≤ 0.005)				
**Affymetrix code**	**Abundance (*pwr-2* / Col)**	**AGI code**	**Name(s)**	**Description**
265560_at	0.4921	At2g05520	GRP3	Encodes a glycine-rich protein that is expressed mainly in stems and leaves
246978_at	0.4936	At5g24910	CYP714A1	member of CYP714A
251621_at	0.4939	At3g57700		Protein kinase superfamily protein
247628_at	0.4954	At5g60400		unknown protein
254265_s_at	0.4962	At4g23140	CRK6	receptor-like protein kinase
246755_at	0.4974	At5g27920		F-box family protein
265414_at	0.5014	At2g16660		Major facilitator superfamily protein
250775_at	0.5043	At5g05460		Glycosyl hydrolase family 85
259560_at	0.5061	At1g21270	WAK2	cytoplasmic serine/threonine protein kinase
266761_at	0.5068	At2g47130		NAD(P)-binding Rossmann-fold superfamily protein
267645_at	0.5133	At2g32860	BGLU33	beta glucosidase 33
249917_at	0.5149	At5g22460		alpha/beta-Hydrolases superfamily protein
245925_at	0.5159	At5g28770	AtbZIP63	bZIP protein BZO2H3
259561_at	0.5238	At1g21250	PRO25, WAK1	cell wall-associated kinase
264529_at	0.5239	At1g30820		CTP synthase family protein
266123_at	0.5242	At2g45180		Bifunctional inhibitor/lipid-transfer protein/seed storage 2S albumin superfamily protein
252387_at	0.5250	At3g47800		Galactose mutarotase-like superfamily protein
263947_at	0.5256	At2g35820		ureidoglycolate hydrolases
261981_at	0.5259	At1g33811		GDSL-motif lipase/hydrolase superfamily protein
260355_at	0.5283	At1g69180	CRC	Putative transcription factor with zinc finger and helix-loop-helix domains, the later similar to HMG boxes. Involved in specifying abaxial cell fate in the carpel.
248551_at	0.5300	At5g50200	WR3	Wound-responsive gene 3 (WR3)
264777_at	0.5302	At1g08630	THA1	Encodes a threonine aldolase
264561_at	0.5373	At1g55810	UKL3	One of the homologous genes predicted to encode proteins with UPRT domains
247162_at	0.5386	At5g65730	XTH6	xyloglucan endotransglucosylase/hydrolase 6
250549_at	0.5386	At5g07860		HXXXD-type acyl-transferase family protein
258845_at	0.5389	At3g03150		unknown protein
254410_at	0.5422	At4g21410	CRK29	Cysteine-rich receptor like protein kinase
253993_at	0.5465	At4g26070	ATMEK1	Member of MAP Kinase Kinase.
244906_at	0.5477	orf240a	ORF240A	Identical to Hypothetical mitochondrial ribosomal S3-like protein AtMg00690 (ORF240a)
257339_s_at	0.5491			
258434_at	0.5509	At3g16770	ATEBP	Encodes a member of the ERF (ethylene response factor) subfamily B-2
254789_at	0.5555	At4g12880		early nodulin-like protein 19 (ENODL19)
253736_at	0.5559	At4g28780		GDSL-like Lipase/Acylhydrolase superfamily protein
262598_at	0.5575	At1g15260		unknown protein
246550_at	0.5584	At5g14920		gibberellin-regulated family protein
260662_at	0.5598	At1g19540		NmrA-like negative transcriptional regulator family protein

**Genes down-regulated in *pwr-2*** (Fold change ≥ 1.5, p-value ≤ 0.005)				
**Affymetrix code**	**Abundance (*pwr-2* / Col)**	**AGI code**	**Name(s)**	**Description**
244943_at	0.5628	nad9	D9	NADH dehydrogenase subunit 9
252117_at	0.5632	At3g51430	SSL5	strictosidine synthase-like protein
247794_at	0.5645	At5g58670	PLC1	phosphatidylinositol-specific phospholipase C
264501_at	0.5657	At1g09390		GDSL-motif lipase/hydrolase family protein
263905_at	0.5661	At2g36190	AtcwINV4	ATCWINV4 (ARABIDOPSIS THALIANA CELL WALL INVERTASE 4)
249245_at	0.5687	At5g42280		Cysteine/Histidine-rich C1 domain family protein
259423_at	0.5692	At1g13880		ELM2 domain-containing protein
260461_at	0.5709	At1g10980		
250613_at	0.5719	At5g07240	IQD24	IQD24 (IQ-domain 24); calmodulin binding
252040_at	0.5727	At3g52060		Core-2/I-branching beta-1,6-N-acetylglucosaminyltransferase family protein
248614_at	0.5732	At5g49560		Putative methyltransferase family protein
248895_at	0.5736	At5g46330	FLS2	Encodes a leucine-rich repeat serine/threonine protein kinase that is expressed ubiquitously.
245117_at	0.5747	At2g41560	ACA4	encodes a calmodulin-regulated Ca(2+)-ATPase
