# Supplementary material for: POWERDRESS and Diversified Expression of the MIR172 Gene Family Bolster the Floral Stem Cell Network
Source: PLoS Genet. 2013 Jan 17;9(1):e1003218. doi: 10.1371/journal.pgen.1003218 (PMC3547843; doi:10.1371/journal.pgen.1003218)
Supplement: Table S2 — Genes up-regulated in pwr-2. (PDF) [file pgen.1003218.s003.pdf]

Table S2. Genes up-regulated in *pwr-2*. Highlighted rows indicate genes discussed in the text.

Genes up-regulated in *pwr-2* (Fold change ≥ 1.5, p-value ≤ 0.005)				
**Affymetrix code**	**Abundance (*pwr-2* / Col)**	**AGI code**	**Name(s)**	**Description**
256977_at	3.7415	At3g21040		copia-like retrotransposon family
250155_at	3.6587	At5g15160	BNQ2	belongs to a family of atypical non-DNA binding basic helix-loop-helix (bHLH). Directly and negatively regulated by AP3 and PI in petals.
256976_at	3.2642	At3g21020		copia-like retrotransposon family
256335_at	3.1118	At1g72110		O-acyltransferase (WSD1-like) family protein
256589_at	2.8024	At3g28740	CYP81D1	cytochrome P450 family protein
264830_at	2.6999	At1g03710		Cystatin/monellin superfamily protein
249920_at	2.6356	At5g19260	FAF3	member of the FAF family proteins encoded by the FANTASTIC FOUR (FAF) genes
264638_at	2.5970	At1g65480	FT	FT, together with LFY, promotes flowering and is antagonistic with its homologous gene, TERMINAL FLOWER1 (TFL1).
249383_at	2.4854	At5g39860	BNQ1, PRE1	Functions downstream of BZR1 to mediate brassinosteroid regulation of cell elongation. BNQ1 is directly and negatively regulated by AP3 and PI in petals.
266532_at	2.1224	At2g16890		UDP-glucoronosyl/UDP-glucosyl transferase family protein
256569_at	2.1001	At3g19550		unknown protein
264363_at	2.0052	At1g03170	FAF2	member of the FAF family proteins encoded by the FANTASTIC FOUR (FAF) genes
248953_at	1.9989	At5g45430		protein kinase superfamily protein
256603_at	1.9974	At3g28270		protein of unknown function
246250_at	1.9632	At4g36880	CP1	cysteine proteinase1
267497_at	1.9090	At2g30540		Thioredoxin superfamily protein
254490_at	1.8740	At4g20320		CTP synthase family protein
254663_at	1.8649	At4g18290	KAT2	member of the Shaker family potassium ion (K+) channel
257136_at	1.8484	At3g12890	ASML2	encodes a protein belonging to a class of CCT (CONSTANS, CONSTANS-like, TOC1) domain proteins
249774_at	1.8403	At5g24150	SQE5, SQP1	squalene monooxygenase gene homolog
251015_at	1.8248	At5g02480		HSP20-like chaperones superfamily protein
247377_at	1.7714	At5g63180		pectate lyase superfamily protein
247351_at	1.7486	At5g63790	AC102	encodes a member of the NAC family of transcription factors
259680_at	1.7350	At1g77690	LAX3	Encodes an auxin influx carrier LAX3 (Like Aux1) that promotes lateral root emergence
253064_at	1.7288	At4g37730	AtbZIP7	bZIP transcription factor family protein
266951_at	1.7090	At2g18940		pentatricopeptide (PPR) repeat-containing protein
253496_at	1.7077	At4g31870	ATGPX7, GPX7	encodes glutathione peroxidase
258338_at	1.7064	At3g16150		N-terminal nucleophile aminohydrolases (Ntn hydrolases) superfamily protein
253252_at	1.7046	At4g34740	ATASE2, CIA1	encodes glutamine 5-phosphoribosylpyrophosphate amidotransferase

Genes up-regulated in *pwr-2* (Fold change ≥ 1.5, p-value ≤ 0.005)				
**Affymetrix code**	**Abundance (*pwr-2* / Col)**	**AGI code**	**Name(s)**	**Description**
264041_at	1.6994	At2g03710	AGL3, SEP4	This gene belongs to the family of SEP genes. It is involved in the development of sepals, petals, stamens and carpels. Additionally, it plays a central role in the determination of flower meristem and organ identity.
251984_at	1.6796	At3g53260	ATPAL2, PAL2	encodes phenylalanine lyase
253880_at	1.6796	At4g27590		heavy metal transport/detoxification superfamily protein
267595_at	1.6615	At2g32990	AtGH9B8	glycosyl hydrolase family 9 protein
267509_at	1.6577	At2g45660	AGL20, SOC1	Controls flowering and is required for CO to promote flowering. It acts downstream of FT.
264002_at	1.6559	At2g22360		DNAJ heat shock family protein
264280_at	1.6512	At1g61820	BGLU46	beta glucosidase 46 (BGLU46)
252863_at	1.6426	At4g39800	MIPS1	Columbia myo-inositol-1-phosphate synthase mRNA
245456_at	1.6405	At4g16950	RPP5	contains a putative nucleotide binding site and leucine-rich repeats
262981_at	1.6337	At1g75590		SAUR-like auxin-responsive protein family
251759_at	1.6311	At3g55630	ATDFD, ATDFD	Encodes one of the three folylpolyglutamate synthetase isoforms
249007_at	1.6311	At5g44650		encodes a nucleus-encoded thylakoid protein
249596_s_at	1.6309	At5g37950		UDP-Glycosyltransferase superfamily protein
245819_at	1.6242	At1g26310	AGL10, CAL	Floral homeotic gene encoding a MADS domain protein homologous to AP1.
253617_at	1.6241	At4g30410		sequence-specific DNA binding transcription factors
247488_at	1.6216	At5g61820		unknown protein
264141_at	1.6189	At1g79090		unknown protein
252652_at	1.6182	At3g44720	ADT4	encodes a plastid-localized arogenate dehydratase involved in phenylalanine biosynthesis
250013_at	1.6114	At5g18040		unknown protein
246844_at	1.6074	At4g38620	ATMYB4, ATMYB86	MYB gene that encodes a transcriptional repressor of the C4H gene which is involved in the biosynthesis of sinapate esters
250185_at	1.6004	At5g14310	AtCXE16	carboxyesterase16
251668_at	1.5961	At3g57010		calcium-dependent phosphotriesterase superfamily protein
247345_at	1.5897	At5g63760	ARI15	functions in zinc-ion binding
260831_at	1.5863	At1g06830		glutaredoxin family protein
257285_at	1.5856	At3g29760		Haloacid dehalogenase-like hydrolase (HAD) superfamily protein
263612_at	1.5807	At2g16440	MCM4	MINICHROMOSOME MAINTENANCE 4 (MCM4); functions in nucleoside-triphosphatase activity
258295_at	1.5805	At3g23400	FIB4	Encodes FIBRILLIN 4 (FIB4). The fibrillins are a large family of chloroplast proteins that have been linked with stress tolerance and disease resistance.
253495_at	1.5718	At4g31850	PGR3	encodes a protein containing 27 pentatrico-peptide repeat (PPR) motifs
250312_at	1.5673	At5g12260		similar to glycosyltransferase family protein 2

Genes up-regulated in *pwr-2* (Fold change ≥ 1.5, p-value ≤ 0.005)				
**Affymetrix code**	**Abundance (*pwr-2* / Col)**	**AGI code**	**Name(s)**	**Description**
266760_at	1.5657	At2g46870	NGA1	NGA1 (NGATHA1); transcription factor
247754_at	1.5561	At5g59080		unknown protein
253052_at	1.5551	At4g37310	CYP81H1	member of CYP81H
248261_at	1.5550	At5g53280	PDV1	integral outer envelope membrane protein, component of the plastid division machinery
260484_at	1.5524	At1g68360		C2H2 and C2HC zinc fingers superfamily protein
251929_at	1.5515	At3g53920	SIG3, SIGC	Encodes a sigma-like transcription factor, Sigma 3 (SIG3 or SIGC). As a subunit of chloroplast RNA polymerase, SIG3 confers the ability to recognize promoter sequences on the core enzyme.
255149_at	1.5464	At4g08150	BP, BP1, KT1	A member of class I knotted1-like homeobox gene family (together with KNAT2).
257227_at	1.5434	At3g27820	MDAR4	encodes a peroxisome membrane-bound monodehydroascorbate reductase
261225_at	1.5422	At1g20100		unknown protein
262348_at	1.5403	At2g48160		PWWP domain-containing protein
257033_at	1.5384	At3g19170	ATZNMP	Zinc metalloprotease pitrilysin subfamily A. Signal peptide degrading enzyme targeted to mitochondria and chloroplasts
261141_at	1.5362	At1g19740		ATP-dependent protease La (LON) domain-containing protein
264799_at	1.5353	At1g08550	AVDE1, NPQ1	Violaxanthin deepoxidase involved in xanthophyll cycle
258878_at	1.5350	At3g03170		unknown protein
265672_at	1.5323	At2g31980	CYS2	PHYTOCYSTATIN2
267250_at	1.5305	At2g23060		Acyl-CoA N-acyltransferases (NAT) superfamily protein
258452_at	1.5287	At3g22370	AOX1A, ATAOX1A	Encodes an isoform of alternative oxidase that is expressed in rosettes, flowers, and root.
250335_at	1.5270	At5g11650		hydrolase, alpha/beta fold family protein
248042_at	1.5266	At5g55960		
252591_at	1.5201	At3g45600	TET3	Member of TETRASPANIN family
260494_at	1.5021	At2g41820		leucine-rich repeat transmembrane kinase family protein
265630_at	1.5016	At2g27350		OTU-like cysteine protease family protein
