# Supplementary material for: POWERDRESS and Diversified Expression of the MIR172 Gene Family Bolster the Floral Stem Cell Network
Source: PLoS Genet. 2013 Jan 17;9(1):e1003218. doi: 10.1371/journal.pgen.1003218 (PMC3547843; doi:10.1371/journal.pgen.1003218)
Supplement: Table S3 — Sequences of oligonucleotide used in this study. (PDF) [file pgen.1003218.s004.pdf]

Table S3. Oligonucleotide sequences used in the present study.

Primer	5' to 3' Sequence	Purpose	Reference (if applicable)
**AGp1**	GCAATTGATGGGTGAGACGATAGGG	Genotyping *ag-10*	
**ag10_genoR**	ATCGGATTCGGGTAATACTTCTCT		
**PWRgeno-F**	TCTTCTTCAGGAGACGCTAC	Genotyping *pwr-1*	
**PWRgeno-R**	TATTTCGCCAGTCCCACACC		
**EN1_XcmIF**	TAGAAAGAAACCACGTCTCGGGTG	Genotyping *pwr-2*	
**3G52250R1**	GTTTACAAGAACAGATACATGATGG		
**R194geno-F**	CTTATGGGTTTTCTTTTGAGC	Genotyping *miR172d-1*	
**R194geno-R**	AACCACATGATGAAAATGGA		
**EN1_full_CACC**	CACCTCTAAGCACTGTGTTTC	Cloning *PWR*	
**EN1cDNA_NS**	AATGGTGTAGCAGAGGCAGCCACG		
**miR156**	GTGCTCACTCTCTTCTGTCA	Northern blotting	
**miR158**	TGCTTTGTCTACATTTGGGA		
**miR159**	TAGAGCTCCCTTCAATCCAAA		
**miR163**	ATCCGAAGTTCCAAGTCCTCCTTCAA		
**miR164**	TGCACGTGCCCTGCTTCTCCA		
**miR166**	CCCCAATGAATCCTGGTCCGT		
**miR172**	ATGCAGCATCATCAAGATTCT		
**miR173**	GTGATTTCTCTCTGTAAGCGAA		
**miR390**	GGCGCTATCCCTCCTGAGCTT		
**U6**	AGGGGCCCATGCTAATCCTTCCTC		
**AP3_RTF3**	TCAAATCTCTTGATCAG	Real-time and RT-PCR	
**AP3_RTR3**	ATGGTTGGGGTAATAGTGGT		
**CRC_RT1**	CTTTGTCGTCAAACCTCCTGAGA		(Schwab et al., 2006)
**CRC_RT2**	TCACTTCTTCTCACCGAATCCCAAGCC		
**EN1_RT_F**	TGGAGACTTCCGCAGACCTT		
**EN1_RT_R**	GGCACTGGACCGAGAAGATG		
**FT_RT1**	TAAGCAGAGTTGTTGGAGACG		(Nakagawa and Komeda, 2004)
**FT_RT2**	TCTAAAGTCTTCTTCCTCCGCAG		
**PI_RT1**	GATGATTGATTACTGTTGTCCTTCC		(Sarnowski et al., 2005)
**PI_RT2**	ATCATGATCTCTCATCATCATTCCT		
**pri-miR172a-F**	AACTCAAACTCAAACCCAAAC		
**pri-miR172a-R**	CAATCAAAACAAGTCATCTCG		
**pri-miR172b_F**	CGGATTAGGGCGTTAATTACAATG		(Hirsch et al., 2006)
**pri-miR172b_R**	GGTCTCTGGACGAACTATTCTGTA		
**pri172cF**	ACACGAATACTTGATGATTTGTGAAT		
**pri172cR**	AAACACCAAATAGGCTCTTCTTTATC		
**pri172dF**	TAGGGTTAGCATGTTGATGACTTC		
**pri172dR**	CCTCAAGTTATCATATCGGAGGAT		
**mir172e_priF**	AAGATTCACAAGAGATGTGGTTCC		
**mir172e_priR**	TATTGCCAAGAGACAGAAAACAAG		
**pri-miR159a-F**	GGAGCTCTACTTCCATCGTCA		(Kim et al., 2011)
**pri-miR159a-R**	CCACGTTCTCATCAAAACTTT		
**pri-miR166b-F**	ATCATTCTCTTCATCATCACCA		
**pri-miR166b-R**	CCCTCTTTAAATCCTCTTCTTCT		

Primer	5' to 3' Sequence	Purpose	Reference (if applicable)
**pri-miR167a-F**	GAAGCTGCCAGCATGATCTA	Real-time and RT-PCR	(Kim et al., 2011)
**pri-miR167a-R**	GGGTTTATAGAAGGGTGCGA		
**pri-miR173F**	CTTCTTCTCACAAATAAACCCAAAT		
**pri-miR173R**	AAGATCTCTAACATTAAATCATCAG		
**pri-miR319aF**	AGAGAGAGCTTCCTTGAGTCCATTCAC		
**pri-miR319aR**	AGGGAGCTCCCTTCAGTCCAATCAAAGAG		
**N_UBQ5**	GGTGCTAAGAAGAGGAAGAAT		(Yu et al., 2008)
**C_UBQ5**	CTCCTTCTTTCTGGTAAACGT		
**AP2-cleavage-R**	GGTTGGAAGCCATTTGTCTG		
**ACT2-intron-F**	GTTCACTTTGGTTCGATTTC		
**ACT2-R**	GAGTAACCACGCTCTGTCAG		
**polII-C1-F**	AGTTCAATGGAGAGATGTCGAAATATG	Pol II ChIP	
**polII-C1-R**	AAGAGGAAAAGAAAGAGATGGAGAGA		
**MIR172a-pro-F**	TGAGAGAGAGAGAGAGAGAGAGA		
**MIR172a-pro-R**	TCAACTTCTGCCAATCAAAC		
**MIR172b-pro-F**	AGGAGAAAAGCAGTGGGATA		
**MIR172b-pro-R**	CCTTGGATTCGTGAGGTT		
**MIR172c-pro-F**	ATGAAGCGATAAAGAAGAGC		
**MIR172c-pro-R**	ATGCACATCATTGGAGAAAA		
**pri172dF**	TAGGGTTAGCATGTTGATGACTTC		
**pri172dR**	CCTCAAGTTATCATATCGGAGGAT		
**mir172e_priF**	AAGATTCACAAGAGATGTGGTTCC		
**mir172e_priR**	TATTGCCAAGAGACAGAAAACAAG		
**MIR166a-pro-F**	TGGCTCTCTCCACTACTCAA		
**MIR166a-pro-R**	GACAACAGTCCCCTCAAAA		
**MIR167a-pro-F**	CGACCCTTAAACTCTCCATAA		
**MIR167a-pro-R**	ACTTCACCGTAGCAGATCAA		
**CRC-ChIP-F**	GTTACCGTGTCAAGCATTC		
**CRC-ChIP-R**	TCAAGCCGAACATCTCTACT		
